# Three-Dimensional Cell Culture: A Breakthrough *in Vivo*

**DOI:** 10.3390/ijms16035517

**Published:** 2015-03-11

**Authors:** Delphine Antoni, Hélène Burckel, Elodie Josset, Georges Noel

**Affiliations:** 1 Radiotherapy Department, Paul Strauss Cancer Center, 3, rue de la Porte de l’Hôpital, 67065 Strasbourg Cedex, France; E-Mail: gnoel@strasbourg.unicancer.fr; 2 Radiobiology Laboratory, EA 3430, Strasbourg University, Paul Strauss Cancer Center, 3, rue de la Porte de l’Hôpital, 67065 Strasbourg Cedex, France; E-Mails: hburckel@strasbourg.unicancer.fr (H.B.); ejosset@strasbourg.unicancer.fr (E.J.)

**Keywords:** cell culture, three-dimensional cell culture, *in vivo*

## Abstract

Cell culture is an important tool for biological research. Two-dimensional cell culture has been used for some time now, but growing cells in flat layers on plastic surfaces does not accurately model the *in vivo* state. As compared to the two-dimensional case, the three-dimensional (3D) cell culture allows biological cells to grow or interact with their surroundings in all three dimensions thanks to an artificial environment. Cells grown in a 3D model have proven to be more physiologically relevant and showed improvements in several studies of biological mechanisms like: cell number monitoring, viability, morphology, proliferation, differentiation, response to stimuli, migration and invasion of tumor cells into surrounding tissues, angiogenesis stimulation and immune system evasion, drug metabolism, gene expression and protein synthesis, general cell function and *in vivo* relevance. 3D culture models succeed thanks to technological advances, including materials science, cell biology and bioreactor design.

## 1. Introduction

Working in three dimensions has widely improved an increasing number of research projects these past years. Cultured mammalian cells are important tools for providing predictions of drug activity, metabolism and toxicity *in vivo*. Physiological relevance is a key parameter to improve prediction made by cell-based assays. Traditionally, two-dimensional cell culture was used to perform *in vitro* research, but its efficiency has been called into question because the environment is far removed from the *in vivo* state. The three-dimensional (3D) cell culture creates an artificial environment in which biological cells are permitted to grow or interact with its surroundings in all three dimensions. Thus, the 3D model more accurately imitates the *in vivo* cells, compared to the previous flat, unnaturally thin, single layer cells grown on two dimension plastic. Thanks to the production of high fidelity models and the long-term maintenance of tissue provided by the three-dimensional cell culture, this method is now currently being used in a wide range of medical and cellular research projects. In this work, we will describe the properties, principles, applications and limitations of the three-dimensional cell cultures.

## 2. Cell Culture

Cell culture refers to the removal of cells from a tissue before their growth to a favorable artificial environment. The cells may be removed from the tissue directly and disaggregated by enzymatic or mechanical means before culture or they may be derived from a cell line or cell strain established before. Cell culture provides appropriate model systems for studying the standard physiology and biochemistry of cells; the effects of drugs and toxic compounds on the cells, mutagenesis and carcinogenesis; in drug screening and development. It also allows consistent and reproducible results. Therefore, it became one of the major tools used in cellular and molecular biology. Cells grow in an artificial environment which contains a substrate or medium that supplies the essential nutrients (amino acids, carbohydrates, vitamins, minerals), growth factors, hormones, gases (O_2_, CO_2_) and regulates the physico-chemical environment (pH, osmotic pressure, temperature). Most cells are anchorage-dependent and must be cultured while attached to a solid or semi-solid substrate (adherent or monolayer culture), while others can be grown floating in the culture medium (suspension culture).

## 3. Three-Dimensional Cell Culture: A Practical Alternative

Conventional adherent tissue culture involves growing cells on solid flat surfaces as two-dimensional (2D) monolayers. Cells are adhering to an artificial plastic or glass substrate and in contact with other cells only at their periphery. Because oxygen, nutrient or waste gradients are absent, the environment is non-physiologically uniform. The cells are not allowed to pile on top of one another, but are forced into monolayer morphology, which is not natural for all cell types. Establishing co-cultures in a flat dish can increase natural intercellular contact and communication, but the 2D surface still inhibits the capacity for cells to form a multi-dimensional structure. Therefore, cells grown in flat layers on plastic surfaces do not accurately model the *in vivo* cells. For example, thanks to the immortalized tumor lines grown in 2D culture, the knowledge about the mechanism of cancer has been improved but with a 95% drug failure rate, they have proven to be a poor drug development model [1], so this make them unreliable predictors of *in vivo* drug efficacy and toxicity. Creating a third dimension for cell culture is clearly more relevant and has to be considered as a practical alternative [2].

Organisms are three-dimensional arrangements of cells with intricate cell-cell and cell-matrix interactions and complex transport dynamics for nutrients and cells. In the *in vivo* state, the cells are maintained in a chemostatic environment courtesy in a constant fresh supply of nutrients thanks to the removal of waste products via the circulatory system. It is important that the culture environment takes into account the spatial organization of the cell [3,4,5]. Three-dimensional cultures are *in vitro* cultures where immortalized cell lines, stem cells, or explants are placed within hydrogel matrices that mimic *in vivo* cell environments. Cells in 3D, as compared to 2D, show improvements in several studies of basic biological mechanisms, which are summarized in Table 1, like: cell number monitoring, viability, morphology, proliferation, differentiation, response to stimuli, cell-cell communication, migration and invasion of tumor cells into surrounding tissues, angiogenesis stimulation and immune system evasion, drug metabolism, gene expression and protein synthesis, general cell function and *in vivo* relevance. 3D cell cultures provide more accurate depiction of cell polarization since in 2D the cells can only be partially polarized. Moreover, 3D cell cultures have greater stability and longer lifespans than cell cultures in 2D. Also, 3D aggregates can be cultured for longer, at least up to 4 weeks, as compared to almost one week with 2D monolayer culture due to cell confluency. Therefore, they might be more appropriate for long-term studies and surviving cells and for demonstrating long-term effects of the drug. Thanks to the 3D environment, the cells are allowed to grow undisturbed compared to the 2D models, where regular trypsinization is necessary in order to provide them with sufficient nutrients for normal cell growth. Compared to 3D, 2D surfaces result in a homogenous cell population, which leads to an overestimation of the effects determined from *in vitro* cytotoxic screens, resulting in an ineffectiveness of the candidate agents *in vivo*. Therefore, 2D culture does not allow testing the effectiveness of certain candidate compounds such as antiangiogenic and vasculogenic agents prior to *in vivo* strategies and permits almost exclusively to identify antiproliferative candidate agents. The proliferation of tumor cells grown in monolayer cultures is faster than that of 3D spheroids, exhibits different metabolic profiles and typically displays improved sensitivity in treatment such as chemotherapy or radiation therapy. As 3D cell culture allows generating 3D aggregates in a non-turbulent environment, with minimal shear force and continually suspended “freefall” culture, it allows the generation of 3D tissue-like aggregates which display physiologically relevant phenotypes such as lower proliferation rate, hypoxic regions and vasculature. Lastly, through analysis of gene expression, microRNA and metabolic profiles, it has been shown that 3D generating aggregates display a genotype significantly more relevant to *in vivo*, as compared to 2D monolayers cultures [6].

**Table 1:** 3D cell culture: Models, advantages and limitations.

Characteristics	Properties
3D culture models	Whole animals and organotypic explant cultures
	Cell spheroids cultures
	Polarized epithelial cell cultures
	Microcarrier cultures
	Tissues-engineered models
Advantages	Cell number monitoring
	Viability
	Morphology
	Proliferation
	Differentiation
	Response to stimuli
	Cell–cell communication
	Migration of tumor cells into surrounding tissues
	Invasion of tumor cells into surrounding tissues
	Cell polarization
	Angiogenesis stimulation
	Immune system evasion
	Drug metabolism
	Gene expression
	Protein synthesis
	General cell function
	Physiological genotype relevance
	Physiological phenotype relevance
	*In vivo* relevance
Limitations	Reproducibility between batches of biomimetic scaffolds
	Extraction of all cells for analysis with increased size and tortuosity
	Creation of 3D matrices
	Capacity to scale up or down a single 3D format
	Handling of post culturing processing
	Imaging depending on the scaffold size, material transparency and microscope depth
	Performance, sensitivity and compatibility with high-throughput screening instruments
	Optimization for 3D cell culturing of the assays used to determine the cellular response to drug interaction (dose dependent cell viability, cell–cell/cell–matrix interaction, cell migration)
	Control of culture conditions (temperature and pH)

## 4. Principles of Three-Dimensional Cell Culture

Important criteria include the choice of material for the scaffold, the source of cells, and the actual methods of culture, which varies considerably according to the tissue of study. Several culture models can be distinguish: the study of whole animals and organotypic explant cultures, cell spheroids, microcarrier cultures and tissues-engineered models [7]. Models of 3D cell culture are summarized in Table 1.

Whole animal and organotypic explants are principally used in studies where an absolute requirement for tissue-specific information is needed [8]. Animal testing is a form of *in vivo* research, which is often employed over *in vitro* because it is better suited for observing the overall effects of an experiment on a living subject. Concerning cancer researches, cancer is established in rodent models by either surgically implanting tumor cells or creating genetically-engineered animals that spontaneously develop human-like tumors in response to experimental modification of gene expression. However, these animal testings could fail to develop into therapies that translate to improve outcomes for human disease [9,10]. There are also many forms of deadly cancer that currently lack a qualified animal model including brain, kidney and skin [11]. Moreover, nowadays, the use of animals in research, teaching and testing is an important ethical and political issue, because many of these experiments cause pain to the animals involved or reduce their quality of life. Therefore, ethical principles of animal use have been developed and rigorous regulations promulgated by several organizations, including the role of the animal experimenters, the need of appropriate premises and also the origin of animals used in the scientific protocols. Recently, European law defined the conditions for using animals. Principle of replacement has been well described. France published this law in its own juridical procedure on the 1 February 2013 (decree n. 2013-118). Moreover, animal models are expensive and time consuming. Isolated perfused organs and tissue slice models offer the closest *in vitro* models of the *in vivo* state. However, these models are expensive, the organs are relatively little available, and maintaining the tissue’s viability *ex vivo* is difficult. These reasons make animal testing, isolated perfused organs and tissue slice models unsuitable for routine testing purposes. Organotypic 3D cell cultures could provide an attractive alternative to animal models for both ethical and economic reasons.

**Figure 1:**
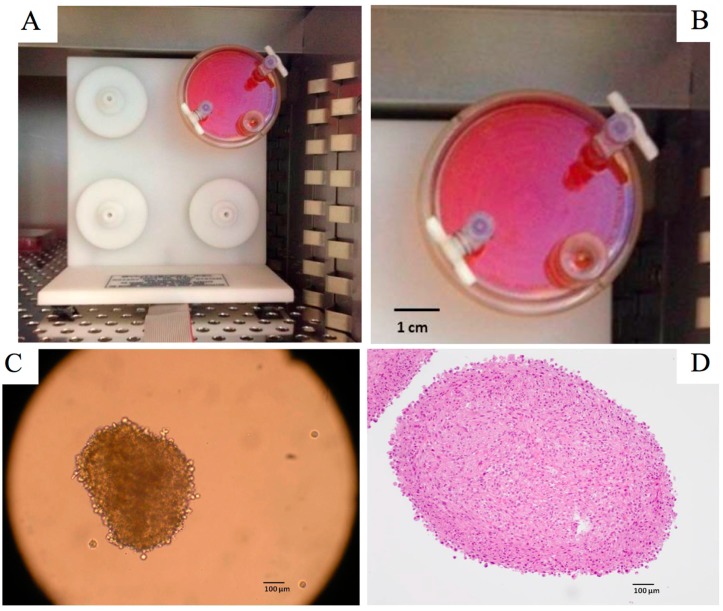
(**A**,**B**) Disposable rotating-wall vessel culture, four station rotator base, Synthecon^®^, Houston, TX, USA; (**C**) Cellular spheroids of glioblastoma cells produced after 6 days by using rotating-wall vessel; (**D**) Tint of a blade of cellular spheroids of glioblastoma cells by the hematoxyline-eosine after 4 weeks culture (Radiobiology Laboratory, EA 3430, Strasbourg, France).

Many cell types have the natural tendency to aggregate. Cells can re-establish mutual contacts and specific microenvironments that allow them to express a tissue-like phenotype. Cellular spheroids represent the most common use of *ex vivo* 3D cultures. They are simple three-dimensional models that can be generated from a wide range of cell types and form due to the tendency of adherent cells to aggregate (Figure 1). Spheroids are self-assembled spherical clusters of cell colonies, created from single culture or co-culture techniques such as hanging drop, rotating culture, or concave plate methods [7,12,13]. The spheroid format is particularly useful in cancer research as it enables quick discovery of morphological changes in transformed cells. Cells are embedded in extracellular matrix (ECM) and left to proliferate and polarize according to the organ of origin. This results in the formation of a perfect sphere if the cells are normal, or a distorted structure if malignant. Extracellular matrices help the cells to be able to move within their spheroid similar to the way cells would move in living tissue. The spheroids are thus improved models for cell migration, differentiation, survival, and growth. The most common types of ECM used are basement membrane extract or collagen. There is also a scaffold-free spheroid culture, where cells are growing suspended in media. This could be achieved either by continue spinning or by using low-adherence plates. No adherence cue is provided to the cells and the culture is largely dependent on cell-cell contacts. Spheroids have seen a use in modeling solid tumor growth and metastasis studies. Spheroids can be harvested and analyzed using colorimetric, fluorescence, and luminescence assays measured with a plate reader. They can also readily be observed by confocal microscopy.

Another approach is the polarized epithelial cell culture [14]. Normal human keratinocytes are isolated from skin and cultured on supports such as collagen or de-epidermalised human dermis which maintains many of the native basement membrane proteins, necessary for adhesion and growth of keratinocytes thereafter [15].

For microcarrier culture, beads derive from dextran, gelatine, glycosaminoglycans and other porous polymers can be used as a 3D support for the culture of anchorage-dependent animal cell lines.

Directional culture is highly suitable for tissue regeneration applications like muscular or neuronal repair and uses native extracellular proteins, such as laminin or collagen, with other matrices such bio-mimetics.

Organotypic cultures involve both an epithelial layer and a mixture of extracellular matrix protein, such as collagen and fibroblasts. They can also use synthetic matrices or artificial scaffolds. Cellular aggregates require the careful exchange of nutrients and gases in addition to spatial control, and problems with cell death arise when aggregate thickness of 1–2 mm arise through a lack of mass transfer, principally through a limited exchange of nutrients and waste metabolites [7,16]. Therefore, the need of scaffolds with a design reflecting the tissue of interest becomes apparent. 3D matrices are available in a large variety of materials with different porosities, pore sizes, permeabilities and mechanical characteristics designed to reflect the *in vivo* ECM of the specific tissues being modeled: metals, ceramics, glasses, natural and synthetic polymers and also composites. The most common materials used are polymers, because the control of their chemical and structural properties, fundamental for dictating the adhesion and spreading of living cells and protein adsorption and in turn cellular attachment [17] are easier to control. Other properties to consider include biocompatibility, wettability, mechanism and methods for fabrication. Due to the variety of material and structural choices for scaffolds, they are widely used in many applications. As the ultimate aim of a scaffold is to produce features found within the ECM required for native cell function, the macro-, micro- and nano-scale elements of 3D scaffolds must be discussed. Macroscale structures are important for determining the overall size and shape of a scaffold, dependent on application. Nano-scale features are important for nutrient supply and functional effects due to the size of many cell-signaling molecules. Microscale must be considered for porosity, pore interconnectivity, pore geometry, pore size, distribution and elements of surface topography. Micro-scale elements may be customized for different tissue types. Micro-scale features facilitate mass transport, diffusion of nutrients, metabolic wastes and soluble molecules and can activate certain genes and modulate cellular behavior in differentiation and proliferation. They also affect the overall robustness of the scaffolds and hence the desired application, such as use in a bioreactor or multi-wells plate.

Bioreactors enable the precise and reproducible control over many environmental conditions required for cell culture, including temperature, pH, medium flow rate, oxygen, nutrient supply, and waste metabolite removal. Bioreactors have been adapted for 3D cell culture and are able to maintain and monitor the environment during growth [18]. Four categories can be identified: rotating wall vessels, direct perfusion systems including hollow fibers, spinner flasks and mechanical force systems. Rotating wall vessels were originally designed by the National Aeronautics and Space Administration to simulate microgravity. They provide continuously moving culture conditions where cell constructs are grown under low shear stress forces and enable high rates of mass transfer [19]. They are composed of two concentric cylinders, an inner stationary cylinder that provides for gas exchange and an outer rotating cylinder. Free-moving scaffolds and media are placed in the space between the two cylinders. Direct perfusion systems allow the culture medium to pass through the construct [20,21]. They use a pump system to perfuse media, directly or indirectly, through a scaffold. The basic design consists of a media reservoir, a pump, a tubing circuit, and a perfusion cartridge, which houses the scaffold. Hollow-fiber systems, a sub-group, are composed of small tube-like filters, approximately 200 μm in diameter with molecular weight cut-offs, sealed into a cartridge shell. Spinner flasks can be used to seed cells into constructs and culture them thereafter. They are composed of a glass media reservoir with side arms that can be opened and may have porous covers for gas exchange. Scaffolds are suspended on a structure or placed within the media, which is stirred by a stir bar. Mechanical force systems exploit the mechanism by which tissues respond to force during growth [22]. Cells such as osteoblasts are known to be mechanoreceptive and respond to force with the activation of intracellular signal transduction pathways [23]. When combined with scaffolds, bioreactors can be used for specialty purposes that utilize mechanical load or electrical or pulsed electrical fields.

3D cell culture in gels can be combined with other models such as spheroid cultures, scaffolds, and microchips. Gels are used as substrates for 3D cell culture. They have a soft tissue-like stiffness and aim to mimic the ECM. They could be made from ECM mixtures of natural origin, such as collagen and alginate. The most commonly used is a reconstituted basement membrane preparation extracted from mouse sarcoma (Matrigel™, Corning Life Sciences, Tewksbury, MA, USA), rich in ECM proteins, such as laminin and collagen, growth factors and enzymes. Gels made from animal sourced natural ECM may vary and change in structure over time therefore synthetic gels, such as the polyethylene glycol based hydrogels, have been developed.

The source of cells for 3D cultures and tissue engineering can be grouped into stem cells, autologous cells, allogenic cells, xenogenic cells, animal-derived primary cells, cell lines, and genetically modified variants of all the above cell types.

## 5. Applications

In February 2010, high-throughput screening technologies (HTS) undertook a survey and report on the use of 3D cell culture. The main applications for the 3D scaffolds by survey respondents have been recorded. These were: investigations of stem cells (64%), also primary cells (61%) and human cell lines (57%), tissue engineering (38%), transformed or recombinant cell lines (36%), cancer cells (35%), growth factor release (21%), angiogenesis (20%), feeder free embryonic stem cells culture (16%), tumor xenografts and other applications (10%). The types of primary cells most investigated by survey respondents for 3D cell culture were fibroblasts, followed by endothelial cells, mesenchymal stem cells and hepatocytes [2]. The 3D scaffolds that have shown greatest promise were gel/hydrogel, followed by ECM sheet, aggregates/spheroids and then collagen tissue constructs. Cell viability, cell proliferation, cell migration and cell signaling were the most successfully improved types of assays with 3D cultures.

Liver cells are often used to test drug toxicity *in vitro*, but the survival rate of primary liver hepatocytes *in vitro* is poor. Using 3D cell culture increases cell viability significantly compared to growth in standard tissue culture plastic and allows cells to be grown in culture for longer periods [24]. Osteoblasts grown with 3D culture remain viable and form complex interactions between adjacent cells. After 21 days in culture, cells secrete extracellular collagen and form bone nodules. Compared to 2D culture, cells grown in 3D produce significantly greater amounts of alkaline phosphatase and osteocalcin. Also, the development of cancer models could be improved by using 3D culture for several reasons. Compare to growing cells in 2D, 3D results in differential zones of proliferation due to oxygen, nutrient and waste gradients [25]. Growing tumor cell lines in 3D induces histological morphology reminiscent of the tumor type from which they were derived. Therefore, 3D culture can be used to study tumor morphology and understand differences between lines from tumors of similar and dissimilar organs and tissues. The cells resistance to chemotherapy can increase in 3D spheroids models. It could be that the cells on the inside of the spheroid are protected from drug penetration by the cells on the outside of the spheroid [26] or that differential proliferation zones exist [27]. 3D cultures recapitulate several mechanisms of drug resistance found in tumors *in vivo*. Moreover, growing cells in 3D allows phenotypic heterogeneity. Gene expression and cell behavior could be also modified when using 3D cell culture. Indeed, differential extracellular interactions with ECM and stromal cells in 3D change intracellular signal transduction were observed, culminating in the activation of a unique set of transcription factors in 2D *versus* 3D models [28]. Growing cells in 3D mimics the tumor microenvironment: cells in 3D have nearly 100% of their surface area exposed to other cells or matrix, whereas cells in 2D have approximately 50% of their surface area exposed to fluid, approximately 50% to the flat culture surface or intermediate, and only a very small percentage to other cells. This is a very important advantage because cancer is a disease of not only the tumor cell but also of the surrounding microenvironment.

## 6. Limitations

While all of the different methods of 3D culture might be a “done deal”, as in easy to use and with improved *in vivo* similarity compared to 2D methods, many problems and unmet needs are remaining (Table 1). Indeed, there is a poor reproducibility between batches of biomimetic scaffolds. Also using scaffolds can make it difficult to extract all cells for analysis with increased size and tortuosity. 3D matrices have too many components and the creation of constructs is difficult and laborious. The capacity to scale up or down a single 3D format and the handling of post culturing processing are limited. Imaging may become difficult depending on the scaffold size, material transparency and microscope depth. Other drawbacks include performance, sensitivity and compatibility with high-throughput screening instruments. The assays used to determine the cellular responses to drug interaction, such as dose dependent cell viability, cell-cell/cell-matrix interaction, and/or cell migration, are not optimized enough for 3D cell culturing. A greater flexibility to accommodate the many different cell-lines and types is needed. 3D cell cultures in gels have also some drawbacks because the culture conditions such as temperature and pH, which must be very carefully controlled, make them often difficult to use. All methods need higher reproducibility and consistence and also improved stability in long-term experiments to be adopted as a routine tool. In the survey from HTS technologies, two-thirds of people surveyed planned to transition their cell culture from 2D to 3D, with half of these having already transitioned some part of their research projects to 3D.

## 7. Conclusions

The availability of accurate, informative *in vitro* assays is an increasingly important challenge for applications in research, toxicity testing and safety screening. Culturing cells in 3D radically enhances cell growth, differentiation and function. 3D culture models can only succeed by combining a number of key areas including materials science, cell biology and bioreactor design. Technological advances have enabled the engineering of 3D scaffolds that offer a real proxy for the *in vivo* environment. Although 3D method has not yet replaced 2D models on a large scale, it has become easier to use and routinely applicable, allowing researchers to switch to this technology in order to obtain better cell culture models.
